# Inhibitor-Sensitive *FGFR1* Amplification in Human Non-Small Cell Lung Cancer

**DOI:** 10.1371/journal.pone.0020351

**Published:** 2011-06-07

**Authors:** Amit Dutt, Alex H. Ramos, Peter S. Hammerman, Craig Mermel, Jeonghee Cho, Tanaz Sharifnia, Ajit Chande, Kumiko Elisa Tanaka, Nicolas Stransky, Heidi Greulich, Nathanael S. Gray, Matthew Meyerson

**Affiliations:** 1 Department of Medical Oncology, Dana-Farber Cancer Institute, Boston, Massachusetts, United States of America; 2 The Broad Institute of Massachusetts Institute of Technology and Harvard, Cambridge, Massachusetts, United States of America; 3 Department of Medicine, Brigham and Women's Hospital and Harvard Medical School, Boston, Massachusetts, United States of America; 4 Department of Cancer Biology, Dana Farber Cancer Institute, Boston, Massachusetts, United States of America; 5 Department of Biological Chemistry and Molecular Pharmacology, Harvard Medical School, Boston, Massachusetts, United States of America; 6 Center for Cancer Genome Discovery, Dana-Farber Cancer Institute, Boston, Massachusetts, United States of America; 7 Department of Pathology, Harvard Medical School, Boston, Massachusetts, United States of America; 8 Advanced Centre for Treatment, Research and Education in Cancer, Tata Memorial Center, Kharghar, Navi Mumbai, India; Medical College of Wisconsin, United States of America

## Abstract

**Background:**

Squamous cell lung carcinomas account for approximately 25% of new lung carcinoma cases and 40,000 deaths per year in the United States. Although there are multiple genomically targeted therapies for lung adenocarcinoma, none has yet been reported in squamous cell lung carcinoma.

**Methodology/Principal Findings:**

Using SNP array analysis, we found that a region of chromosome segment 8p11-12 containing three genes–*WHSC1L1*, *LETM2*, and *FGFR1*–is amplified in 3% of lung adenocarcinomas and 21% of squamous cell lung carcinomas. Furthermore, we demonstrated that a non-small cell lung carcinoma cell line harboring focal amplification of *FGFR1* is dependent on FGFR1 activity for cell growth, as treatment of this cell line either with *FGFR1*-specific shRNAs or with FGFR small molecule enzymatic inhibitors leads to cell growth inhibition.

**Conclusions/Significance:**

These studies show that *FGFR1* amplification is common in squamous cell lung cancer, and that FGFR1 may represent a promising therapeutic target in non-small cell lung cancer.

## Introduction

Lung cancer is the leading cause of cancer-related death in developed countries with deaths in 2009 estimated at approximately 160,000 in the United States, accounting for about 28% of all cancer deaths [1]. Non-small cell lung cancer (NSCLC) accounts for 75% of all lung cancers and includes two predominant subtypes, adenocarcinoma and squamous cell carcinoma (SCC), which comprise 40% and 25% of NSCLCs, respectively [2], [3]. Despite clear histologic and biologic distinctions, lung adenocarcinoma and squamous cell carcinoma are largely treated with the same chemotherapeutic agents with the exception of the antifolate agent pemetrexed which is approved for the treatment of non-squamous NSCLC [4].

Significant advances in the treatment of lung adenocarcinoma have stemmed from detailed genomic analyses and the deployment of molecularly targeted agents leading which have led to improvements in patient outcomes. Examples include the use of epidermal growth factor receptor (EGFR) inhibitors such as gefitinib and erlotinib [5], [6], [7] for lung adenocarcinomas bearing *EGFR* mutations [8], [9], [10], and of ALK inhibitors such as crizotinib [11] for lung adenocarcinomas bearing *EML4-ALK* translocations [12], [13].

However, little is currently known about the targetable genetic abnormalities underlying squamous cell lung cancer. In addition to *TP53* mutations [14], squamous cell lung carcinomas have been shown to harbor amplifications of *PIK3CA* [15], *SOX2* [16], and *EGFR* [16] as well as *EGFR* variant III mutations [17] *DDR2* mutations [18] and rare amplifications of *PDGFRA/KIT* [19], [20] and *BRF2* [21]. A recent study has demonstrated focal amplification of the *FGFR1* locus on chromosome 8p associated with cellular dependency on *FGFR1* and sensitivity to FGFR inhibitors [22]. At this time there are no FDA-approved targeted therapies for squamous cell lung cancer.

Targeting amplified tyrosine kinases with antibodies or with small molecule inhibitors has led to dramatic improvements in response rates and overall survival of cancer patients whose tumors harbor specific genomic abnormalities. Amplifications of *EGFR* and *ERBB2* have been reported in a variety of malignancies, including head and neck, esophageal, gastric, breast and colon cancers as well as NSCLC [23]. Targeting of these tyrosine kinases, such as the use of cetuximab to target *EGFR* in colorectal and head and neck cancer [24], [25] and the use of trastuzumab to target *ERBB2* in breast cancer [26], has resulted in significant improvement in patient outcomes in each of these diseases, though not all patients with these amplifications respond to targeted agents [27], [28], likely due to additional genomic alterations within the tumor that result in primary resistance to specific agents [29], [30].

The fibroblast growth factor receptor type 1 gene (*FGFR1*) is one of the most commonly amplified genes in human cancer [16]. The fibroblast growth factor receptor (FGFR) tyrosine kinase family is comprised of four kinases, FGFR1, 2, 3, and 4, that play crucial role in development, and have been shown to be targets for deregulation by either amplification, point mutation, or translocation (reviewed in [31]). Translocations involving *FGFR3*, as well as activating somatic mutations in *FGFR3* have been identified in multiple myeloma and bladder cancer [32], [33], [34]. We and others have identified activating mutations in *FGFR2* in endometrial cancer [35], [36]. Amplification or activation of *FGFR1* has been reported in oral squamous carcinoma [37], esophageal squamous cell carcinomas [38], ovarian cancer [39], bladder cancer [40], prostate cancer [41], rhabodomyosarcoma [42], and lung cancer [16], [43], [44], [45], [46]. Consistent with this, a pan-FGFR tyrosine kinase inhibitor has been shown to block tumor proliferation in a subset of NSCLC cell lines with activated FGFR signaling but has no effect on cells that do not activate the pathway [47]. *FGFR1* has been identified as the driver event in breast carcinomas and NSCLC, especially squamous cell lung carcinomas, harboring similar amplifications of the 8p11 chromosomal segment [22], [48]

Based on SNP array copy number analysis of 732 samples, we report that *FGFR1* is somatically amplified in 21% of lung squamous cell carcinomas as compared to 3.4% of lung adenocarcinomas. We validate FGFR1 as a potential therapeutic target by showing that at least one *FGFR1*-amplified NSCLC tumor cell line is sensitive to FGFR enzymatic inhibition and dependent on *FGFR1* expression for cell viability as evidenced by shRNA treatment. Together with previous reports reviewed above, these results suggest that FGFR1 may be an attractive therapeutic target in NSCLC.

## Materials and Methods

### NSCLC primary samples and cell lines

NSCLC cell lines, NCI-H1703 (squamous), NCI-H2444 (pulmonary), NCI-H520 (squamous), HCC95 (squamous), NCI-1581 (large cell carcinoma), Calu3 (not otherwise specified), NCI-H1734 (not otherwise specified), Colo699 (adenocarcinoma), NCI-H2170 (squamous), NCI-H226 (squamous), A427 (adenocarcinoma), NCI-H1563 (adenocarcinoma), NCI-H1781 (adenocarcinoma) and HCC15 (squamous) were obtained from the collection of A.F. Gazdar, J. Minna, and colleagues [49], [50], [51], from ATCC (Manassas, Virginia, United States) and/or DSMZ (Braunschweig, Germany). Cells were maintained in RPMI 1640 complete media supplemented with 10% calf serum (Gibco/Invitrogen, Carlsbad, California, United States) and penicillin/streptomycin (Gibco/Invitrogen). The NSCLC tumor/normal pairs analyzed in this study have been described earlier [16], [20], [45], [50], [52].

### SNP array data analysis

SNP array experiments were performed on 732 NSCLC tumor and cell line samples and data analyzed as described previously [16], [20], [45], [50], [52]. The boundaries of the 8p11 amplicon defined by GISTIC analysis [53] were identified as reported [52](http://www.broadinstitute.org/tumorscape/pages/portalHome.jsf). Data display has been performed using the Integrative Genomics Viewer (http://www.broadinstitute.org/igv).

### Transfection and infection

Phoenix 293T packaging cell line (Orbigen, San Diego, California, United States) were transfected with pBabe-Puro-based gateway vectors using FuGENE® 6 Transfection Reagent (Roche, Indianapolis, United States) to generate replication incompetent retroviruses. Target cells were infected with these retroviruses in the presence of 8 µg/ml polybrene. Two days post infection, cells were treated with 2 µg/ml puromycin (Sigma, St. Louis, Missouri, United States) for two days. The resulting stable cell lines were used for experimental studies.

### shRNA mediated *FGFR1* knockdown

shRNA vectors were obtained from TRC (The RNAi Consortium). The target sequences of the shRNA constructs are:

*FGFR1*#1 (TRCN 0000121307): 5′- AGTGGCTTATTAATTCCGATA-3′.

*FGFR1* #2 (TRCN 0000121308): 5′- GCTTGCCAATGGCGGACTCAA-3′.

*FGFR1* #3 (TRCN 0000121309): 5′- CTTGTATGTCATCGTGGAGTA-3′.

*FGFR1* #4 (TRCN 0000121310): 5′- CAAGATGAAGAGTGGTACCAA-3′.

*FGFR1* #5 (TRCN 0000121311): 5′- GAATGAGTACGGCAGCATCAA-3′.

*LETM2* #1 (TRCN 0000040243): 5′- CGCACCTTCTACCTGATAGAT-3′.

*LETM2* #2 (TRCN 0000040244): 5′- CCCAGCACAAAGGAGATAGTT-3′.

*LETM2* #3 (TRCN 0000040245): 5′- CCAGTTACATCATCACCCATA-3′.

*LETM2* #4 (TRCN 0000040246): 5′- CCAGGAACTAGACTAATACAA-3′.

*LETM2* #4 (TRCN 0000040247): 5′- GCATTGAGTGTATCAGAACTA-3′.

*WHSC1L1*#1 (TRCN 0000015613): 5′- CGAGAGTATAAAGGTCATAAA-3′.

*WHSC1L1* #2 (TRCN 0000015614): 5′- CCATCATCAATCAGTGTGTAT-3′.

*WHSC1L1* #3 (TRCN 0000015615): 5′- CGAGAATATCATGTCCAGTTT-3′.

*WHSC1L1* #4 (TRCN 0000015616): 5′- GCTTCCATTACGATGCACAAA-3′.

*WHSC1L1* #5 (TRCN 0000015617): 5′- GCAGGGAATTGTTTGAGTCTT-3′.

The sequence targeted by the *GFP* shRNA is 5′-GCAAGCTGACCCTGAAGTTCAT-3′. Lentiviruses were made by transfection of 293T packaging cells with these constructs using a three-plasmid system as previously described [54]. Target cells were incubated with lentiviruses for 6 hours in the presence of 8 µg/ml polybrene and left in fresh media. Cells were grown for two days. Fifty micrograms of total cell lysates prepared from the infected cell lines was analyzed by Western blotting.

### Growth and Proliferation assays

For survival assays, 2×10^6^ cells for each tumor cell line expressing shRNAs constructs targeting *FGFR1*, *WHSC1L1*, *LETM2* or *GFP* along with uninfected cells were seeded in 3 replicates on a 6 well plate. Cell viability was determined at 24 hour time points for 4 consecutive days by counting the cells using Beckman Coulter Vi-Cell Automated Cell Viability Analyzer following trypan blue dye staining. The percentage of cell viability was plotted for each cell line of readings obtained on day 4 relative to day 1.

### Soft agar anchorage-independent growth assay

For soft agar assays, 2×10^4^ NSCLC cells expressing sh*FGFR1* and sh*GFP* were suspended in a top layer of RPMI1640 containing 10% calf serum and 0.4% Select agar (Gibco/Invitrogen, Carlsbad, California, United States) and plated on a bottom layer of RPMI1640 containing 10% calf serum and 0.5% Select agar on a 6 well plate. PD173074 or FIIN-1 [55] was added as described to the top agar. After 3–5 weeks incubation colonies were counted in triplicate. IC50s were determined by nonlinear regression using Prism 5 software (GraphPad Software).

### Cytotoxicity assays

Depending on the growth curves for each cell line, between 800 and 2000 NSCLC cells were seeded in 6 replicates in 96- well plate. One day after plating, increasing doses of FGFR inhibitors PD173074 or FIIN-1 were added and proliferation of cells was assessed 4 days later using the WST-1 assay (Roche Applied Science). Each data point represents the median of six replicate wells for each tumor cell line and inhibitor concentration. IC50s were determined by nonlinear regression using the Prism Graphpad software.

### Western Blots

Total protein was extracted and separated by gel electrophoresis by lysing cells in a buffer containing 50 mM Tris-HCl (pH 7.4), 150 mM NaCl, 2.5 mM EDTA, 1% Triton X-100, and 0.25% IGEPAL. Protease inhibitors (Roche Applied Science) and phosphatase inhibitors (Calbiochem) were added prior to use. Before loading to the gel, samples were normalized for total protein content. Total protein lysates were boiled in sample buffer, separated by SDS-PAGE on 8% polyacrylamide gels, transferred to PVDF membrane, and probed overnight using the appropriate primary antibodies. Antibodies used for immunoblotting were: anti- FGFR1 antibody (# 3472, Cell Signaling Technologies, Danvers, MA, United States), anti- phospho FRS2 Y436 (#3861, Cell Signaling Technologies, Danvers, MA, United States), anti-phospho-FRS2 Y196 (#3864, Cell Signaling Technologies, Danvers, MA, United States), anti-FRS2 (# sc-17841, Santa Cruz Biotechnology, Santa Cruz, CA, United States). anti-WHSC1L1 monoclonal antibody (# sc-130009, Santa Cruz Biotechnology), anti-LETM2 monoclonal antibody (# ab84626, Abcam), and anti-Actin monoclonal antibody (# sc-1615, Santa Cruz Biotechnology).

### Statistical Analysis

Comparisons between SNP array copy number data for lung adenocarcinoma (AC) and squamous cell carcinoma (SCC) tumors were performed using Fisher's exact T test to calculate two-tailed p-values among samples harboring high level amplification, defined as log2 ratio >0.7 or 3.25 normalized DNA copies. P values<0.05 were considered significant.

To determine the IC50 for FGFR inhibitors, the cell viability measurements of six replicates at varying concentrations of inhibitors were normalized to untreated control cells. Sigmoidal dose response curves were fitted to the data by non linear regression using GraphPad Prism software. Standard deviations were determined for the mean of each value using an inbuilt module of the software.

For shRNA experiments, performed in three replicates, the cell number was counted and the mean and standard deviation were determined using functions in Microsoft Excel.

## Results

### *FGFR1* is amplified in non-small cell lung cancer

**Figure 1:**
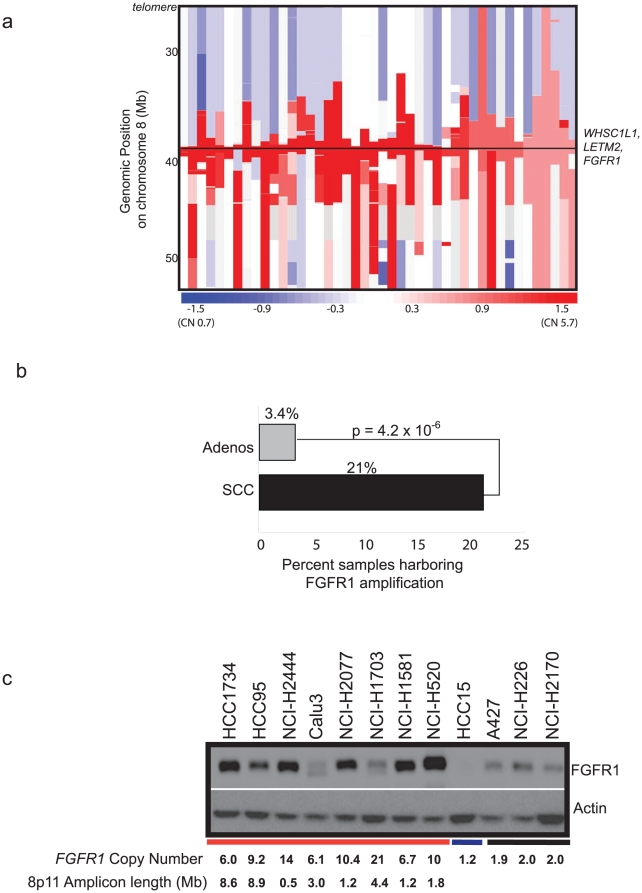
Amplifications of *FGFR1* locus in NSCLC. (A) Copy number estimates at chromosome arm 8p11-12q for 44 NSCLC samples (columns; ordered by amplification of 8p11) having amplification greater than 3.25 copies (log2 ratio of 0.7) from a collection of 732 NSCLC primary samples and cell lines. The horizontal line indicates the region containing *FGFR1*, *LETM2* and *WHSC1L1* genes. The color scale ranges from blue (deletion) to red (amplification) with estimated copy numbers shown. Grey regions represent the absence of SNP copy number data. (B) Bar graph depicting percentages of samples harboring 8p11-12 amplification in lung adenocarcinomas (AC) and squamous cell carcinoma (SCC) demonstrates that *FGFR1* amplification is observed in SCC at much higher frequency than AC. (C) FGFR1 expression (upper panel) shown in ten NSCLC cells; eight cell lines harboring *FGFR1* amplification—HCC1734, HCC95, NCI-H2444, Calu3, NCI-H2077, NCI-H1703, NCI-H1581 and NCI-H520 (indicated by red horizontal bar below)—one NSCLC cell line harboring deletion of the region HCC15 (indicated by blue horizontal bar below)– and three NSCLC cells with no amplification—A427, NCI-H226, NCI-H2170 (indicated by black horizontal bar below)– using actin as a loading control (shown in lower panel). *FGFR1* copy number status and 8p11-12 amplicon length determined by SNP array is indicated below cells harboring amplification. Of note, NCI-H2077 and NCI-1581 were found to be genotypically identical by fingerprinting analysis.

We examined the 8p11-12 genomic region using Affymetrix 250K SNP array copy number data in a previously reported data set of 732 NSCLC samples, (628 primary tumors and 104 cell lines) (Table S1) [16], [20], [45], [50], [52]. We observed high level amplification, defined as log2 ratio >0.7 or 3.25 normalized DNA copies, of the 8p11-12 chromosomal segment encompassing the *FGFR1* locus in 44 (6%) of NSCLC samples (Figure 1a; Table S2). The majority (93%; 41/44) of these amplifications were relatively focal events (<50% of the length of chromosome 8p) indicating preferential selection of the specific target genes within the region of amplification [52]. The inferred copy number of the amplifications, normalized to a copy number of 2 for each sample, ranged from 3.25 to 25 copies (median = 2.8 copies). The estimated extent of the region of focal amplifications ranged from 0.47 to 112.7 Mb (median = 2.74 Mb).

To identify regions of significant copy-number alteration, we applied GISTIC (Genomic Identification of Significant Targets In Cancer) [53], and identified a 170 Kb region on 8p11 (38.28 to 38.45 Mb) as significantly amplified. While the overall pattern of 8p11 amplification was consistent with the literature on lung cancer as reported, our sample size and resolution provided more power to accurately identify and localize both large-scale and focal chromosomal alterations as compared to earlier reports [45], [52]. The sole genes within the region of amplification identified in our analysis across all samples were *FGFR1* and *LETM2*. In our copy number data, *WHSC1L1* was generally amplified with *FGFR1* and *LETM2* (41/44 samples) but the whole *WHSC1L1* gene did not fall within the GISTIC peak (chr8:38,284,229–38,451,475). Specifically, three primary tumor samples with amplified *FGFR1* and *LETM2* genes had amplicon breakpoints within *WHSC1L1*, explaining the exclusion of *WHSC1L1* from the GISTIC amplification peak (Figure S1; Figure S2). The amplicon breakpoints within *WHSC1L1* are consistent with a lack of amplification of the functional SET domain (chr8:38,265,630–38,255,125) associated with histone methyltransferase enzymatic activity of the gene product [56]. These results do not exclude *WHSC1L1* as the target of amplification on 8p11 along with *FGFR1* but suggest that its histone methyltransferase activity is not likely to be specifically targeted for amplification.

In comparing subtypes of NSCLC primary tumors and cell lines, 3.4% (20/588) of adenocarcinomas and 21% (12/57) of squamous cell carcinomas harbored 8p11 amplifications, indicating that while 8p11 is amplified at appreciable frequencies across both major NSCLC subtypes, it is preferentially amplified in SCCs (*p*<0.001, Fisher exact test) (Figure 1b). No statistically significant correlations were observed between the presence of 8p11 amplifications and available clinical parameters including histology, degree of histological differentiation, stage at surgical resection of the tumors and the age, gender, or reported ethnicity of the patients (Table S3). Additionally, targeted sequencing of the kinase domain of *FGFR1* in 52 NSCLC cell lines and of the entire *FGFR1* coding sequence in three cell lines (NCI-H1581, NCI-H1703, NCI-H2170) did not reveal any evidence of kinase domain mutations (data not shown).

The SNP array data revealed an elevated *FGFR1* gene copy number in 11 NSCLC cell lines out of 104 NSCLC cell lines analyzed (Figure S3) with amplifications observed in 36% (4/11) of squamous NSCLC cell lines assayed. We examined FGFR1 protein expression by immunoblot analysis in 8 primary NSCLC cell lines that harbor focal or broad 8p11 amplification above a log2 ratio of 1.6 or 6.0 normalized DNA copies (NCI-H1703, NCI-H2444, NCI-H520, NCI-1581, NCH-H2077, Calu3, NCI-H1734, and HCC 95), 3 that have approximately neutral *FGFR1* copy number (NCI-2170, NCI-H226 and A427) and 3 that harbor *FGFR1* deletion (NCI-H1781, NCI-H1563 and HCC15) by immunoblot analysis. We found 6 out of 8 *FGFR1* amplified NSCLC cell lines overexpress FGFR1 as compared to cell lines that do not harbor amplification, with the exceptions of the NCI-H1703 and Calu3 cells (Figure S4; Figure 1c). Consistent with this finding, NCI-H1703, which harbors a 8p11 amplification, has been shown not to be dependent on *FGFR1* [44] but on amplified *PDGFRA* [19], [20]. Furthermore, an elevated level of phosphorylation of the FGFR1 substrate FRS2 was observed in NCI-H1581 large cell carcinoma cells carrying focal amplification of *FGFR1*, but not in cells harboring relatively broader levels of *FGFR1* amplification (Figure S4).

### *FGFR1* is required for survival of an NSCLC cell line harboring focal amplification

Based on our copy number analysis, *FGFR1* and *LETM2* fell within the GISTIC-defined region of statistically significant amplification with *WHSC11* immediately adjacent. To determine the cellular requirement for genes in the region targeted by the amplification, we assessed the requirement of *WHSC1L1*, *LETM2* and *FGFR1* expression for tumor maintenance by depleting them individually using shRNA. Transfection with five shRNA constructs targeting either *WHSC1L1* or *LETM2* had no differential effect on the survival of cells harboring focal or broad 8p11-12 amplification as compared to control cells without the amplification (data not shown).

**Figure 2:**
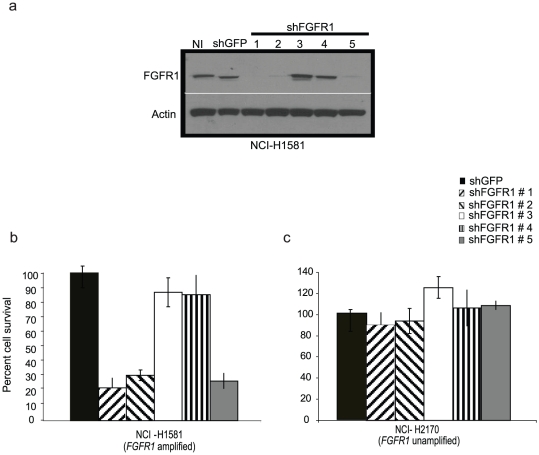
NCI-H1581 cells are sensitive to knock-down of FGFR1 expression. (A) Effects of five *FGFR1* shRNA constructs on FGFR1 protein expression in NCI-H1581 cells as assayed by immunoblotting. shRNAs #1, #2 and #5 efficiently knock down endogenous FGFR1 expression in NCI-H1581 cells infected with shRNA-expressing lentiviruses while shRNAs #3 and #4 do not. Actin is shown as a loading control (lower panel). *(B* and *C)*, infection with three independent FGFR1-suppressing hairpins (#1, #2 and #5) inhibits survival of NCI-H1581 cells over expressing *FGFR1* (B) but did not inhibit survival of cells not harboring *FGFR1* amplification, NCI-H2170 (C) as assessed by WST assay. NI, no infection. shGFP, control hairpin specific for green fluorescent protein used as a negative control. All results normalized to survival of cells infected with shGFP.

In contrast, three out of five shRNA constructs targeting *FGFR1*, all of which led to a 3- to 5-fold decrease in FGFR1 protein levels relative to shRNA controls (Figure 2a), significantly inhibited cell survival in an NSCLC cell line carrying a focal *FGFR1* amplification (NCI-H1581; Figure 2b). shRNA constructs #3 and #4, which did not lead to significant knockdown of FGFR1 protein levels, did not affect the survival of cells harboring 8p11 amplification (Figure 2b). There were no observed survival effects of *FGFR1* shRNA on cell lines harboring relatively broader *FGFR1* amplification (NCI-H1703, HCC95, HCC1734, Calu3; not shown) or without *FGFR1* amplification (NCI-H2170; Figure 2c. HCC15, NCI-H1563 and NCI-H1781; not shown). Overall, these results argue that *FGFR1* expression is required for the viability of at least one NSCLC cell line carrying an *FGFR1* amplification.

**Figure 3:**
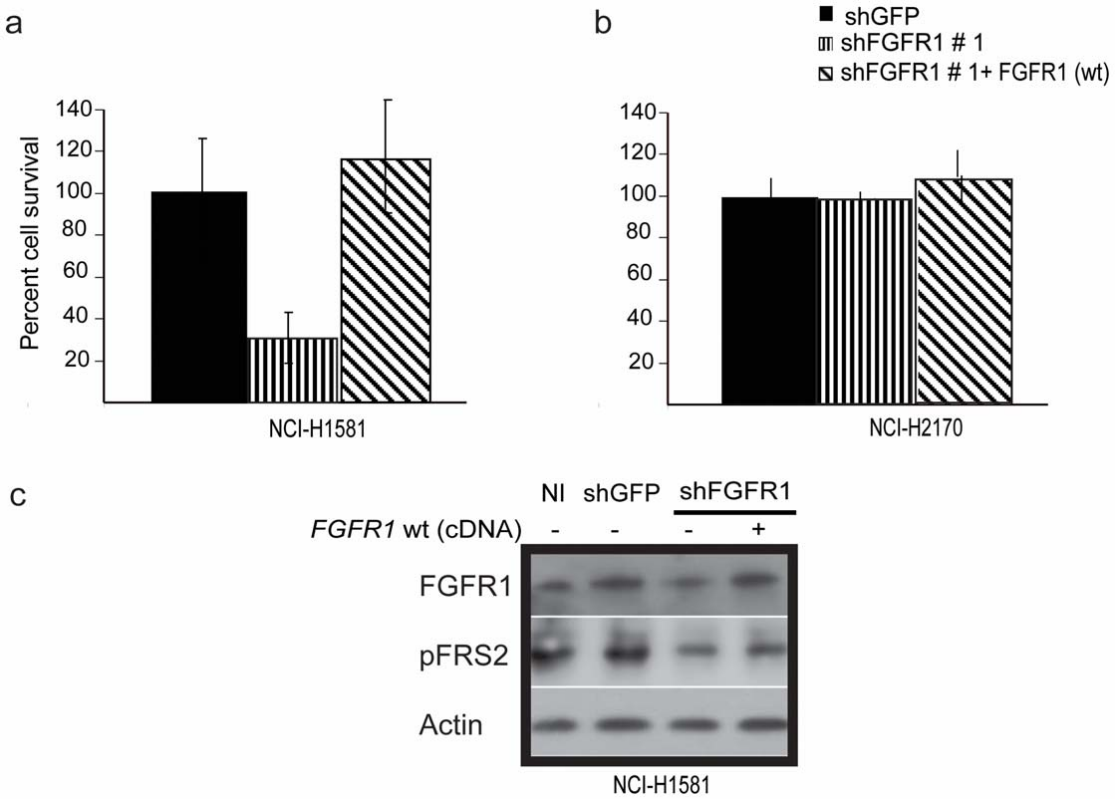
Ectopic expression of *FGFR1* coding region rescues lethality of an shRNA targeting the *FGFR1* 3′ UTR. (A) Bar graph for rescue assay. Lethality due to depleted levels on endogenous FGFR1 level in NCI-H1581 is rescued by over expression of wild type (Wt) full length *FGFR1* coding sequence . (B) No effect on the survival of NCI-H2170 cells was observed due to over expression of wild type form of FGFR1. NCI-H2170 is not dependent on FGFR1 activity. NI, no infection. shGFP, control hairpin specific for green fluorescent protein used as a negative control. All results are normalized to survival of cells infected with shGFP. Data shown is as mean of three replicates. (C) Validation of FGFR1 rescue by immunoblotting. Depleted levels of endogenous FGFR1 level in NCI-H1581 cells infected with *FGFR1* shRNA-expressing lentiviruses targeting the FGFR1 3′UTR (lane 1) is rescued by overexpression of wild type form of FGFR1 cDNA lacking the 3′UTR (lane 2) with concomitant modest rescue in the levels tyrosine residue phosphorylation of the FGFR1 substrate FRS2 (middle panel). Actin is shown as a loading control (lower panel).

To further validate the specificity of cell viability changes associated with shRNA-induced FGFR1-depletion, we examined the ability of ectopic expression of *FGFR1* cDNA to rescue the effects of FGFR1 knock down. Wild-type *FGFR1* cDNA, lacking the 3′-untranslated region (UTR) of the endogenous FGFR1 mRNA targeted by FGFR1 shRNA #1, was over-expressed in NCI-H1581 cells transfected with this shRNA construct. Reconstituted levels of wild type FGFR1 protein resulted in significant rescue of the survival inhibition phenotype (Figure 3a, c) but had no impact on the *FGFR1*-independent NCI-H2170 cells (Figure 3b). Collectively, these experiments implicate *FGFR1* as a critical oncogenic target of 8p11-12 amplification.

Interestingly, one NSCLC cell line carrying a focal *FGFR1* amplification, NCI-H2444 (Figure S3), was not sensitive to knockdown of FGFR1 (data not shown). This cell line also harbors an activating *KRAS* G12V mutation [57], [58], which is associated with resistance of colorectal cancers to the EGFR-directed therapy cetuximab [25]. NCI-H2444 does not show FRS2 phosphorylation (Figure S4). This observation suggests that co-occurrence of other activating oncogenes may relieve *FGFR1* dependence and specifically that primary resistance to FGFR1 inhibition may be governed by *KRAS* mutation status.

### FGFR kinase Inhibitors inhibits growth of *FGFR1* amplified NSCLC cells

**Figure 4:**
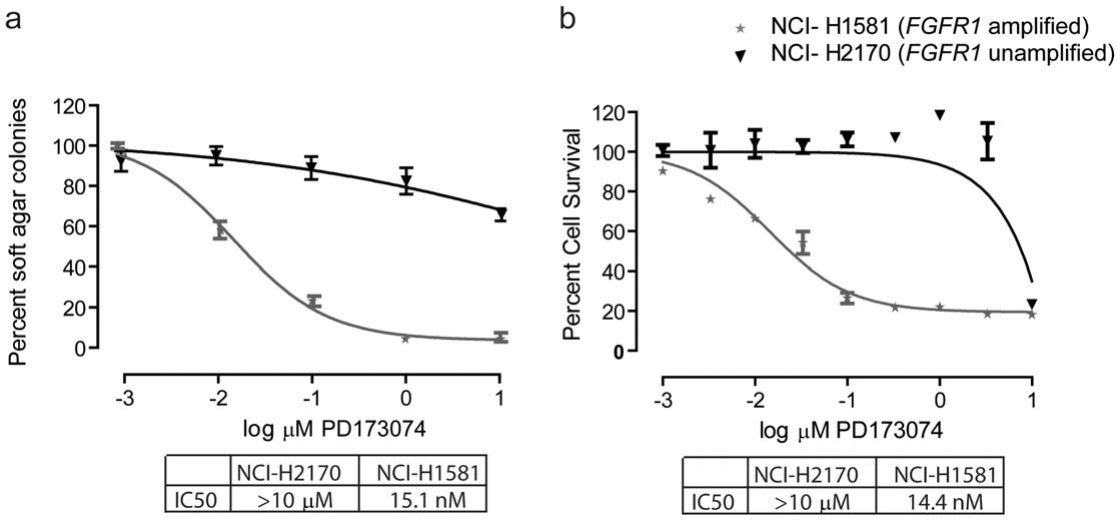
FGFR1 tyrosine kinase activity is essential in NCI-H1581 cells. (A) Treatment with the indicated concentrations of pan FGFR inhibitor PD173074 inhibited soft agar colony formation by the NCI-H1581 NSCLC cell lines harboring *FGFR1* amplification, as compared with the NCI-H2170 line, which does not harbor *FGFR1* amplification. Colonies were photographed and quantitated after 4 weeks. (B) Treatment with the indicated concentrations of PD173074 inhibited survival of NCI-H1581 cells, but not of NCI-H2170 cells, as determined by WST assay performed after 4 days treatment. IC50s are indicated.

To evaluate the possibility that targeting *FGFR1* in 8p11 amplified SCCs could represent a new therapeutic strategy in SCCs, we studied the effects of the pan-FGFR inhibitor PD173074 on NSCLC cell lines. The *FGFR1*-amplified NCI-H1581 cells were sensitive to treatment with PD173074 as assayed by colony formation in soft agar with IC_50_s in the range of 10–20 nM (Figure 4a and Figure S5). In contrast, NCI-H2170 cells with wild type *FGFR1* copy number were insensitive to PD173074 (Figure 4a). We also performed PD173074 dose response curves on cell survival in liquid culture to compare the sensitivity of cells harboring *FGFR1* amplification and those without and again found that NCI-H1581 cells were killed at IC50 values of 14 nM, while those without amplification required more than 100-fold higher doses of PD173074 to inhibit proliferation (Figure 4b). In agreement with these results, we also observed that a second FGFR irreversible inhibitor, FIIN-1, inhibits proliferation of NCI-H1581 cells with focal *FGFR1* amplification as compared to NCI-H2170 without *FGFR1* amplification, with IC50 values of 2.5 nM versus greater than 10 micromolar, respectively (Figure S6).

## Discussion

Here we have shown that *FGFR1* is frequently amplified in lung carcinomas and that this amplification is enriched in lung SCCs. At least one NSCLC cell line with focally amplified *FGFR1* requires the gene as demonstrated by shRNA depletion, and is also sensitive to inhibition with FGFR kinase inhibitors.

Genes other than *FGFR1* have been proposed to be the functional target of amplification on chromosome segment 8p11-8p12, most notably *WHSC1L1* [44] and *BRF2* [21]. However, we believe that the evidence presented here as well as in a recent report [22] argues for *FGFR1* as the functional target of amplification in at least one NSCLC cell line. Additionally, in our data set *WHSC1L1* is not amplified in all the *FGFR1* amplified samples, arguing that it is unlikely to be the only relevant amplified gene in the 8p11-12 amplicon. The cell line that was shown to require *WHSC1L1* for its survival, NCI-H1703 [44], does not over-express FGFR1 (Figure 1c), does not show FRS2 phosphorylation (Figure S4) and is dependent on another amplified tyrosine kinase oncogene, *PDGFRA* [19], [20]. In contrast, knockdown of *WHSC1L1* had no impact on *FGFR1*-amplified, *FGFR1*-expressing NCI-H1581 cells, suggesting that amplification of either gene may contribute to cellular transformation in the appropriate cellular context.

A recent study characterizing DNA amplification in NSCLC suggested that *BRF2*, encoding a transcription initiation complex subunit of RNA polymerase III, is the target of amplification in the 8p11 amplicon [21]. We compared *FGFR1* amplification to *BRF2* amplification in light of this report and found that of 12 samples with the highest amplification of *FGFR1* in our dataset (log2 ratio >2.5), only 4 samples include *BRF2* in the amplified region, suggesting that *BRF2* is not the predominant target of 8p11 amplification in SCC (Figure S7a). We also found that of the 12 samples with highest amplification of *BRF2* (log 2 ratio >1.8), all have *FGFR1* amplification (in one case, with what appears to be a translocation within FGFR1) (Figure S7b). We believe that these data argue in favor of *FGFR1* instead of *BRF2* as the more commonly amplified gene in this region.

Our study and a recent report [22] identify FGFR1 as a potential therapeutic target in NSCLC, where 8p11-12 amplification is common, suggesting that high levels of expression of *FGFR1* may contribute to tumorigenesis or progression in NSCLC. Interestingly, we did not find evidence of *FGFR1* mutation in 52 samples which argues in favor of amplification rather than mutation being the preferred mechanism of *FGFR1* activation in a subset of NSCLCs. As *FGFR1* amplification has been reported in other tumor types, it may be the case that FGFR1 inhibition will be a successful therapeutic strategy in a variety of settings. As several FGFR kinase inhibitors are now in clinical trials, including brivanib [59], dovitinib [60], BIBF 1120 [61], and SU-6668 [62], it could be useful to test these inhibitors on NSCLC patients bearing focal *FGFR1* amplifications. Given that our results suggest that amplification alone will not always predict sensitivity to FGFR1 inhibition, additional work is needed to fully characterize the genetic alterations involved in NSCLC carcinogenesis and dependency on FGFR1.

## Supporting Information

Figure S1 ***WHSC1L1*** **histone methyltransferase activity domain is not likely to be specifically targeted for amplification at 8p11-12q.** Heat map representation of SNP array based segmented copy number on chromosome arm 8p11-12q for 34 NSCLC samples (rows; ordered by amplification of a 170 kb chromosomal segment spanning *WHSC1L1*, *LETM2* and *FGFR1*) having amplification greater than 3.25 copies (log2 ratio of 0.7) from a collection of 732 NSCLC primary samples and cell lines. Three primary tumor samples marked * harbor amplicon with breakpoints within *WHSC1L1* and only amplify *FGFR1* and *LETM2* genes. The sample marked **#** appears to be a translocation within *FGFR1* removing its first exon. The locations of WHSC1L1 SET domain and FGFR1 kinase domain are indicated. The color scale ranges from blue (deletion) to red (amplification) with estimated copy numbers as shown. Grey denotes a region for which no SNPs are present on the array and therefore represents indeterminate copy number.
(TIF)

Figure S2 **Exclusion of** ***WHSC1L1*** **functional domain among primary tumors harboring amplified** ***FGFR1*** **and** ***LETM2*** **.** Bar graph representation of unsegmented probe-level copy number values for amplicons in 3 primary tumor samples harboring break points within *WHSC1L1*. Estimated copy number values (y axis) are plotted for individual SNPs at 8p11-12 locus (x axis). Copy number of SNPs defining boundary of breakpoint are indicated. Genomic positions of genes in region are shown along the x axis.
(TIF)

Figure S3 **Elevated** ***FGFR1*** **gene copy number in NSCLC cell lines.** SNP array based segmented copy number on chromosome arm 8p11-12q for 18 NSCLC cell lines (rows; ordered by amplification) from telomere (left) to centromere (right). The color scale ranges from blue (deletion) to red (amplification) with estimated copy numbers shown.
(TIF)

Figure S4 **Activation of FGFR1 substrate FRS2 in NCI-H1581 cells.** Western blot analysis of FGFR1 in five different 8p11-12 amplified cells (Colo699, Calu3, NCI-H2077, NCIH1581, NCI-H520 and NCIH1703) indicated by red horizontal bar below and in three NSCLC cell lines harboring deletion of the region (NCI-H1781, NCI-H1563 and HCC15) indicated by blue horizontal bar below. NCI-H1581 cells show increased tyrosine residue phosphorylation of FGFR1 substrate FRS2 as compared to other NSCLC cell lines using actin as a loading control (shown in lower panel).
(TIF)

Figure S5 **FGFR1 tyrosine kinase activity is essential for NCI-H1581 anchorage independent growth.** Inhibition of soft agar colony formation by the NCI-H1581 NSCLC cell line harboring *FGFR1* amplification, in the presence of increasing concentrations of FGFR inhibitor PD173074, compared with HCC15 and NCI-H2170 cells without *FGFR1* amplification, and NCI-H1703 cells that harbor *FGFR1* amplification but do not over-express FGFR1. Cells were seeded in soft agar and treated with different concentrations of PD173074. Representative plates from two independent experiments are presented. Colonies were photographed and quantitated after 4 weeks.
(TIF)

Figure S6 **FGFR1 tyrosine kinase activity is essential in proliferation of NCI-H1581 cells.** Treatment with the indicated concentrations of irreversible FGFR inhibitor FIIN-1 inhibited survival of NCI-H1581 cells, but not of NCI-H2170 cells, as determined by WST assay performed after 4 days treatment. IC50s are indicated.
(TIF)

Figure S7 ***FGFR1*** **instead of** ***BRF2*** **is the more commonly amplified gene at 8p11.** Copy-number data from chromosome 8p11-12 in 12 samples sorted by highest copy number on the top. The view is sorted by *FGFR1* amplification (A) and *BRF2* amplification (B). (A) Of the 12 samples with highest amplification at *FGFR1* of log2 ratio above 2.5, only 4 samples amplify *BRF2* at similar levels. (B) Out of 12 samples with log_2_ ratio above 1.8 at *BRF2*, all samples include *FGFR1* amplification. Each sample is represented as a horizontal row from telomere (left) to telomere (right). Areas of red indicate gain; blue indicates loss. The positions of *FGFR1* and *BRF2* are indicated with vertical lines.
(TIF)

Table S1 **List of NSCLC Samples Analyzed by SNP Array**.
(XLS)

Table S2 **Amplicons at 8p11-12 overlapping** ***WHSC1L1, LETM2 and FGFR1***.
(XLS)

Table S3 **Categorization of 628 Primary Samples by 8p11 Amplification Status and Clinical Features**.
(XLS)
