# Supplementary figures and images for: Inhibitor-Sensitive FGFR1 Amplification in Human Non-Small Cell Lung Cancer
Source: PLoS One. 2011 Jun 7;6(6):e20351. doi: 10.1371/journal.pone.0020351 (PMC3110189; doi:10.1371/journal.pone.0020351)

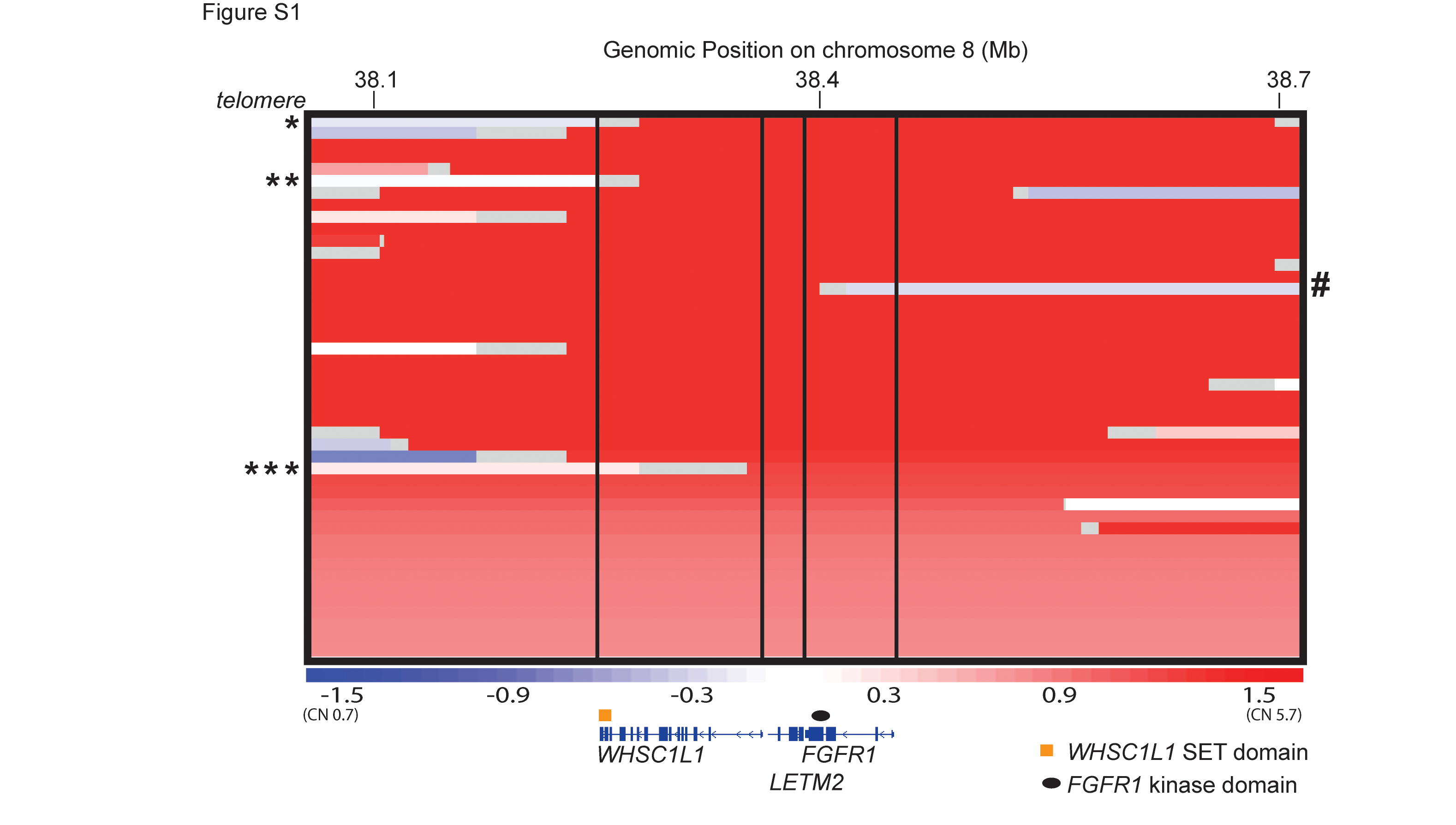

Supplement: Figure S1 — WHSC1L1 histone methyltransferase activity domain is not likely to be specifically targeted for amplification at 8p11-12q. Heat map representation of SNP array based segmented copy number on chromosome arm 8p11-12q for 34 NSCLC samples (rows; ordered by amplification of a 170 kb chromosomal segment spanning WHSC1L1, LETM2 and FGFR1) having amplification greater than 3.25 copies (log2 ratio of 0.7) from a collection of 732 NSCLC primary samples and cell lines. Three primary tumor samples marked * harbor amplicon with breakpoints within WHSC1L1 and only amplify FGFR1 and LETM2 genes. The sample marked # appears to be a translocation within FGFR1 removing its first exon. The locations of WHSC1L1 SET domain and FGFR1 kinase domain are indicated. The color scale ranges from blue (deletion) to red (amplification) with estimated copy numbers as shown. Grey denotes a region for which no SNPs are present on the array and therefore represents indeterminate copy number. (TIF) [file pone.0020351.s001.tif]

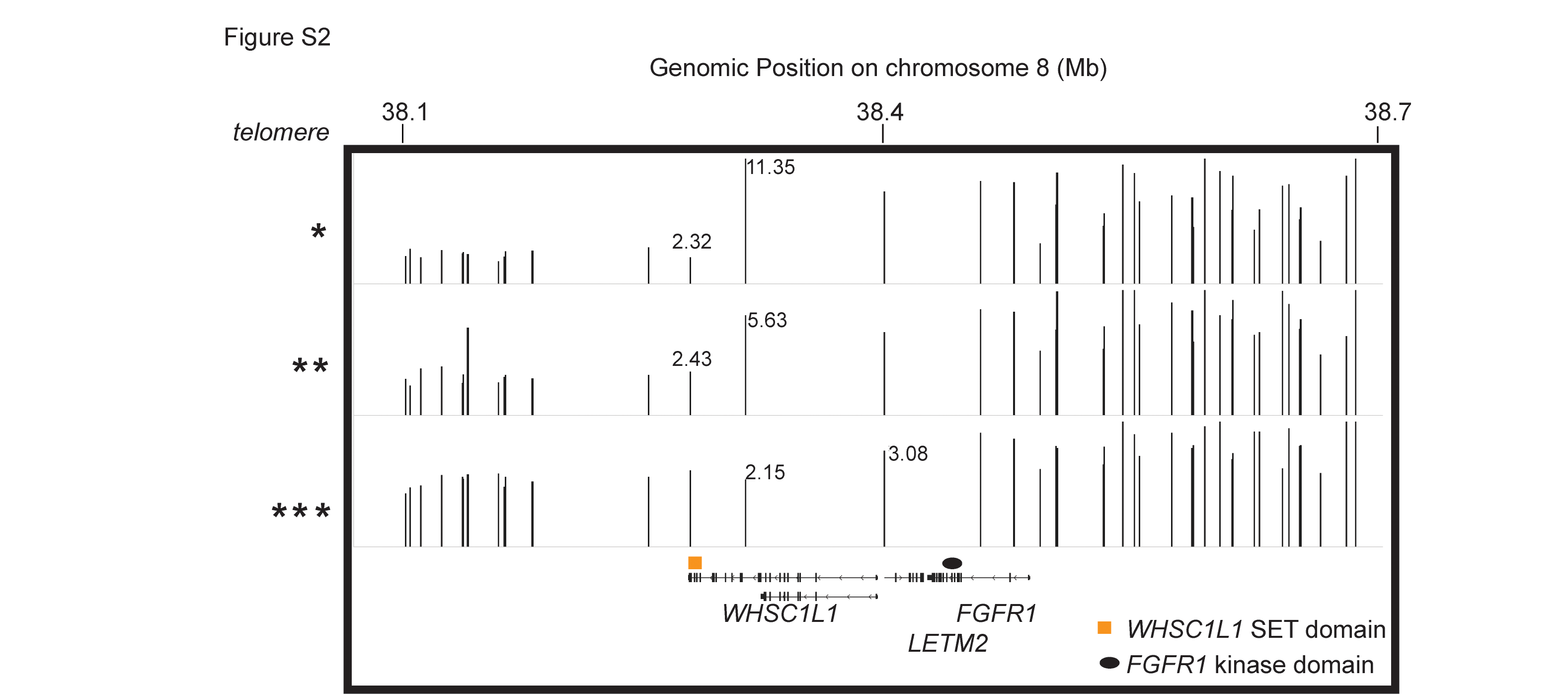

Supplement: Figure S2 — Exclusion of WHSC1L1 functional domain among primary tumors harboring amplified FGFR1 and LETM2 . Bar graph representation of unsegmented probe-level copy number values for amplicons in 3 primary tumor samples harboring break points within WHSC1L1. Estimated copy number values (y axis) are plotted for individual SNPs at 8p11-12 locus (x axis). Copy number of SNPs defining boundary of breakpoint are indicated. Genomic positions of genes in region are shown along the x axis. (TIF) [file pone.0020351.s002.tif]

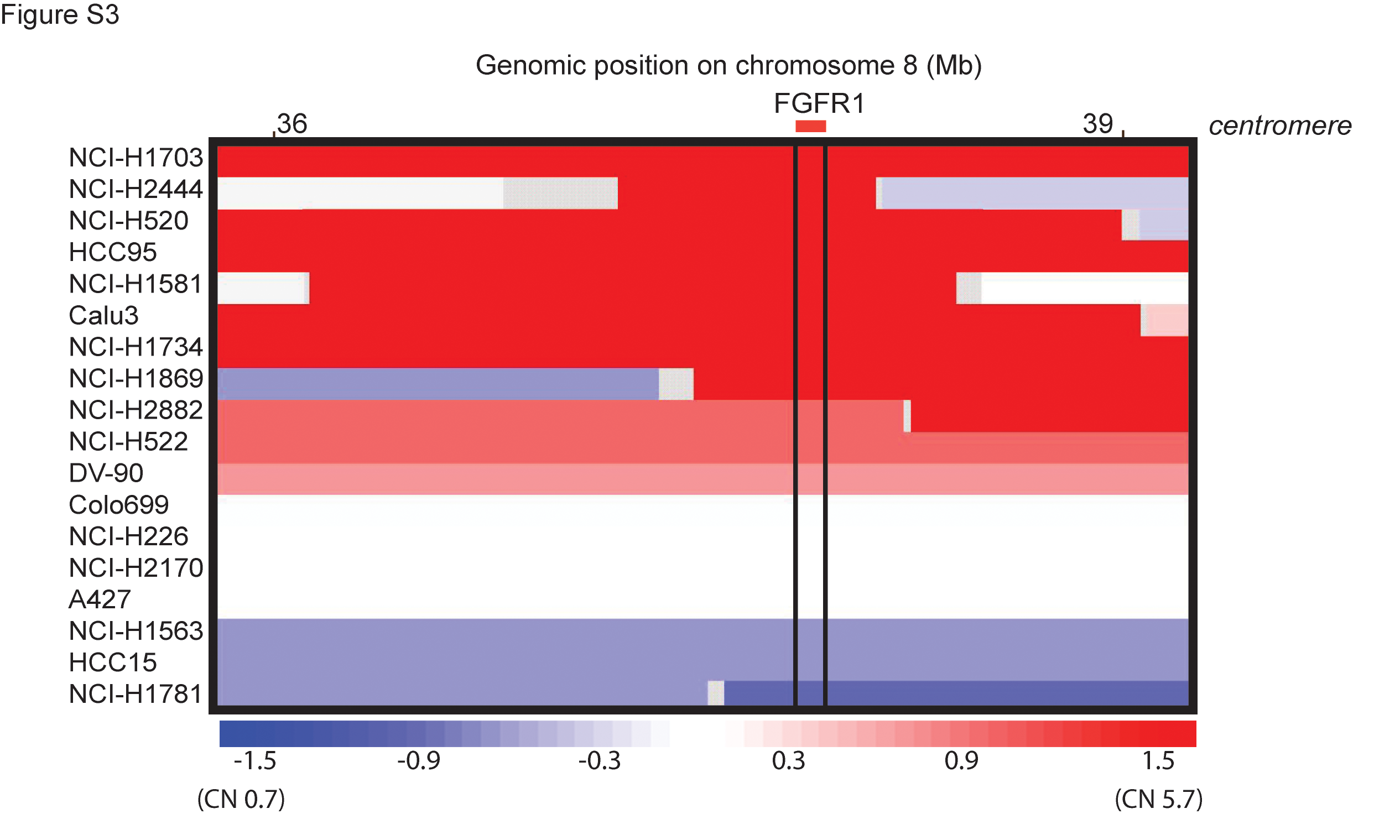

Supplement: Figure S3 — Elevated FGFR1 gene copy number in NSCLC cell lines. SNP array based segmented copy number on chromosome arm 8p11-12q for 18 NSCLC cell lines (rows; ordered by amplification) from telomere (left) to centromere (right). The color scale ranges from blue (deletion) to red (amplification) with estimated copy numbers shown. (TIF) [file pone.0020351.s003.tif]

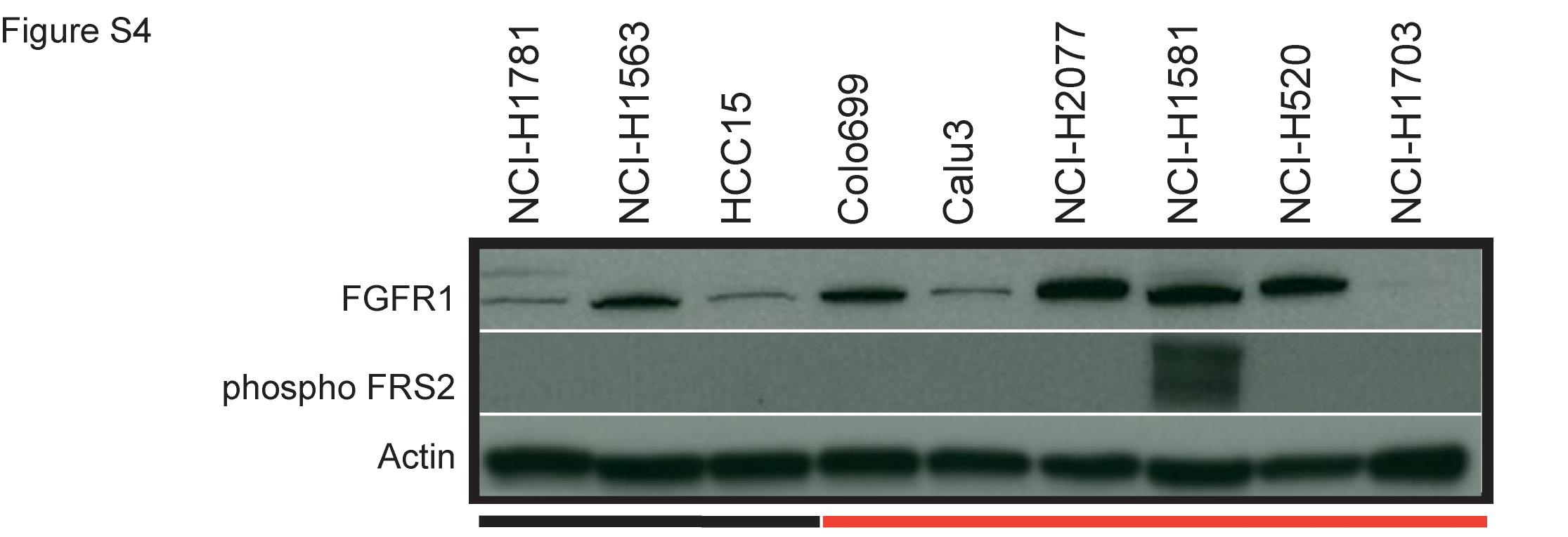

Supplement: Figure S4 — Activation of FGFR1 substrate FRS2 in NCI-H1581 cells. Western blot analysis of FGFR1 in five different 8p11-12 amplified cells (Colo699, Calu3, NCI-H2077, NCIH1581, NCI-H520 and NCIH1703) indicated by red horizontal bar below and in three NSCLC cell lines harboring deletion of the region (NCI-H1781, NCI-H1563 and HCC15) indicated by blue horizontal bar below. NCI-H1581 cells show increased tyrosine residue phosphorylation of FGFR1 substrate FRS2 as compared to other NSCLC cell lines using actin as a loading control (shown in lower panel). (TIF) [file pone.0020351.s004.tif]

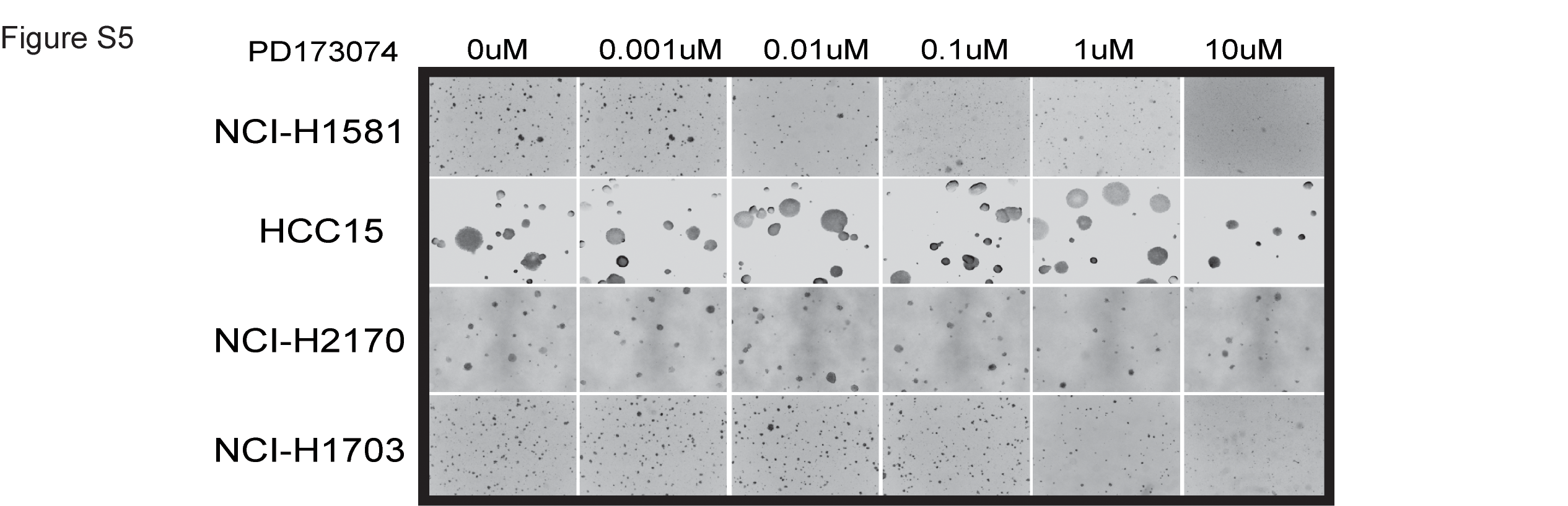

Supplement: Figure S5 — FGFR1 tyrosine kinase activity is essential for NCI-H1581 anchorage independent growth. Inhibition of soft agar colony formation by the NCI-H1581 NSCLC cell line harboring FGFR1 amplification, in the presence of increasing concentrations of FGFR inhibitor PD173074, compared with HCC15 and NCI-H2170 cells without FGFR1 amplification, and NCI-H1703 cells that harbor FGFR1 amplification but do not over-express FGFR1. Cells were seeded in soft agar and treated with different concentrations of PD173074. Representative plates from two independent experiments are presented. Colonies were photographed and quantitated after 4 weeks. (TIF) [file pone.0020351.s005.tif]

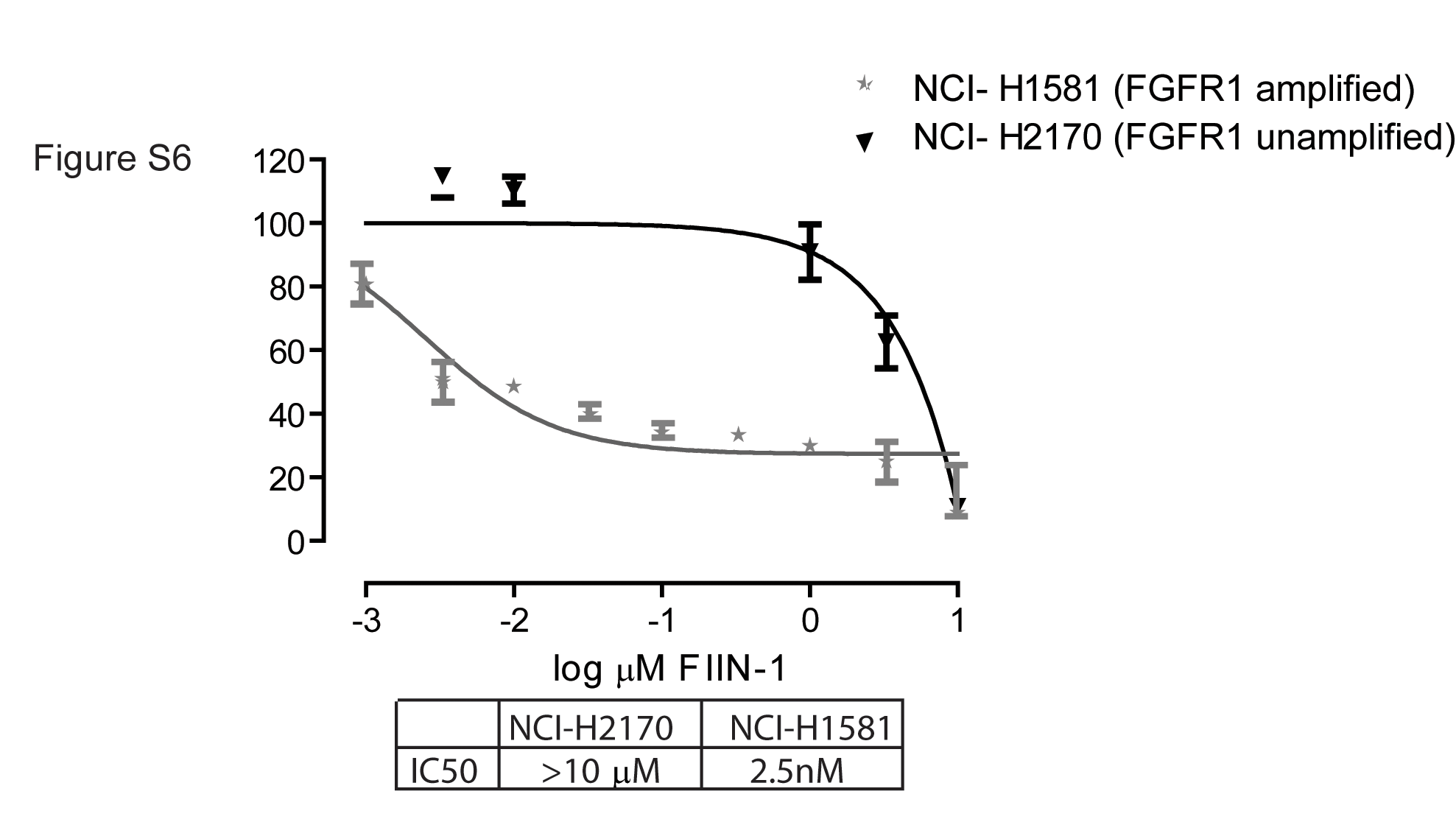

Supplement: Figure S6 — FGFR1 tyrosine kinase activity is essential in proliferation of NCI-H1581 cells. Treatment with the indicated concentrations of irreversible FGFR inhibitor FIIN-1 inhibited survival of NCI-H1581 cells, but not of NCI-H2170 cells, as determined by WST assay performed after 4 days treatment. IC50s are indicated. (TIF) [file pone.0020351.s006.tif]

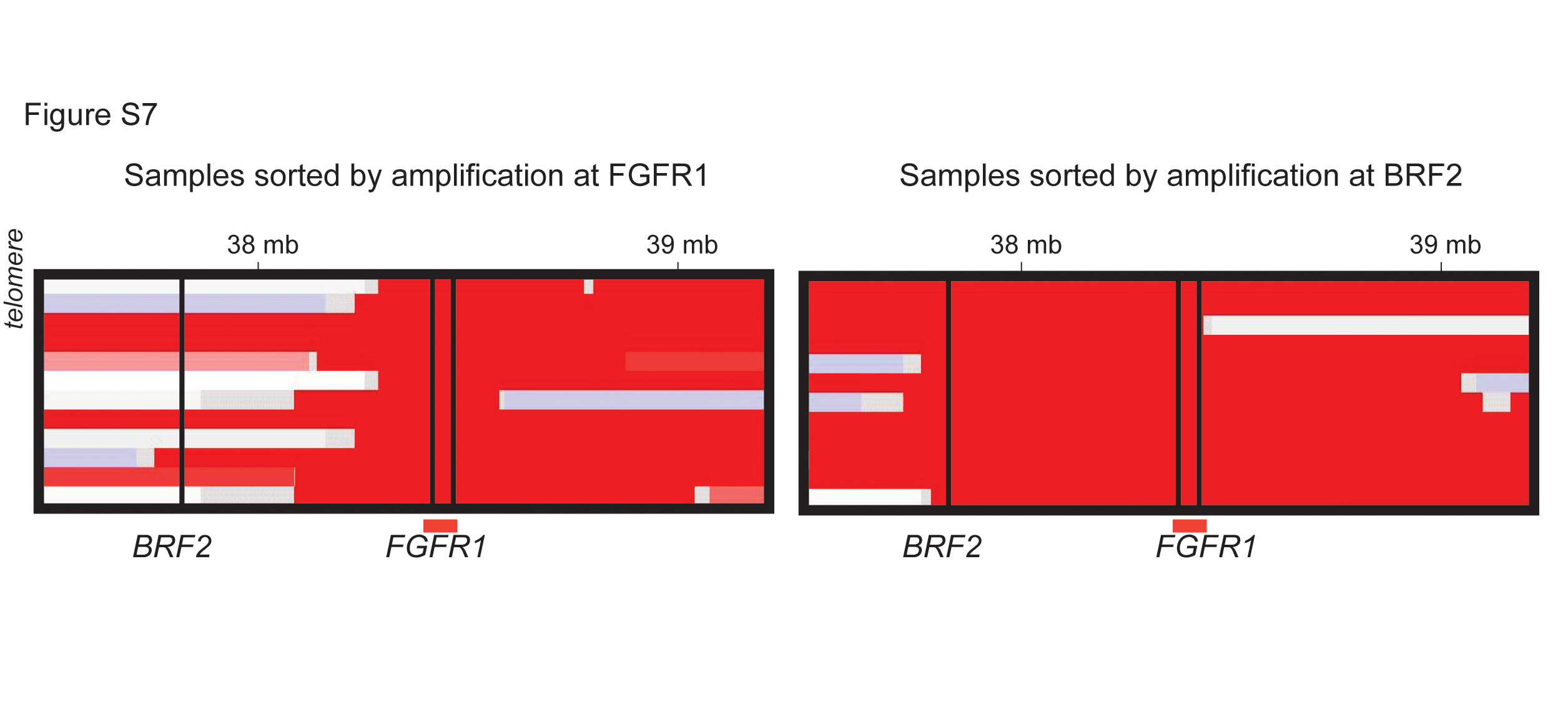

Supplement: Figure S7 — FGFR1 instead of BRF2 is the more commonly amplified gene at 8p11. Copy-number data from chromosome 8p11-12 in 12 samples sorted by highest copy number on the top. The view is sorted by FGFR1 amplification (A) and BRF2 amplification (B). (A) Of the 12 samples with highest amplification at FGFR1 of log2 ratio above 2.5, only 4 samples amplify BRF2 at similar levels. (B) Out of 12 samples with log2 ratio above 1.8 at BRF2, all samples include FGFR1 amplification. Each sample is represented as a horizontal row from telomere (left) to telomere (right). Areas of red indicate gain; blue indicates loss. The positions of FGFR1 and BRF2 are indicated with vertical lines. (TIF) [file pone.0020351.s007.tif]
